# Mechanistic Pathways of Pesticide-Induced Intestinal Injury: Oxidative Stress, Inflammation, and Barrier Dysfunction

**DOI:** 10.3390/cimb48101083

**Published:** 2026-10-20

**Authors:** Nathalia Kirsten, Shayane Milhorini da Silva, Camila Bach de Assis, Salvador Paganella Chaves Júnior, Izonete Cristina Guiloski, Daniele Maria-Ferreira

**Affiliations:** 1 Faculdades Pequeno Príncipe, Av. Iguaçu, 333, Rebouças, Curitiba CEP 80230-020, PR, Braziliguiloski@gmail.com (I.C.G.); 2 Instituto de Pesquisa Pelé Pequeno Príncipe, Av. Silva Jardim, 1632, Água Verde, Curitiba CEP 80250-060, PR, Brazil

**Keywords:** pesticides, intestinal toxicity, oxidative stress, epithelial barrier, inflammation, intestinal permeability

## Abstract

Pesticides are among the most widely used environmental contaminants worldwide, resulting in continuous human exposure primarily through dietary intake. As a major site of contact following oral exposure, the gastrointestinal tract represents a relevant target for pesticide-associated toxicity. Although accumulating evidence indicates that pesticides can alter intestinal structure and function, the toxicological mechanisms underlying these effects have not been critically integrated. This narrative review evaluates experimental studies in rodent models investigating the intestinal toxicity of herbicides, insecticides, and fungicides, with particular emphasis on the convergence and heterogeneity of the reported mechanisms. Across the studies reviewed, recurrent alterations included oxidative imbalance, inflammatory activation, epithelial barrier disruption, changes in intestinal permeability, mitochondrial dysfunction, and genotoxicity. However, the occurrence and magnitude of these responses varied according to pesticide class, compound, dose, exposure duration, formulation, and experimental model, and the available evidence does not support a universal mechanistic sequence. By comparatively integrating these findings, this review highlights shared and pesticide-specific intestinal responses while distinguishing recurrent associations from established causal relationships. Important knowledge gaps remain regarding chronic low-dose exposure, pesticide mixtures, sex-specific susceptibility, recovery after exposure, and the translational relevance of experimental findings to humans. Overall, this review provides a critical framework for interpreting pesticide-associated intestinal toxicity and identifies priorities for future experimental and translational research.

## 1. Introduction

Pesticides are widely used worldwide to enhance agricultural productivity and support food security, and their global use continues to rise [1]. In 2022, total pesticide consumption reached 3.70 million tons of active ingredients (Mt), a 4% increase compared to 2021 [2]. Although pesticides play an essential role in modern agriculture, growing concern has emerged about the unintended consequences of chronic environmental and dietary exposure [1]. Pesticide residues have been detected in soil, water, and food, resulting in exposure of non-target organisms, including humans [1,3]. Epidemiological and experimental studies have linked both direct and indirect pesticide exposure to a wide range of adverse health outcomes, including diabetes, neurological and reproductive disorders, cancer, and other chronic diseases [4,5,6]. However, the extent to which these associations reflect direct causal effects depends on the specific pesticide, exposure conditions, and available evidence.

In non-occupational settings, exposure in the general population occurs primarily through ingestion of pesticide-contaminated food and drinking water [3]. As the primary route of exposure for the general population, oral ingestion makes the gastrointestinal tract the first site of interaction with pesticide residues and their metabolites. Unlike other target organs, intestinal tissues are directly exposed to parent compounds and their metabolites before systemic distribution, making the intestine an important site of toxicological interaction. Increasing evidence suggests that pesticide exposure can compromise the integrity of the intestinal epithelial barrier, an early and pivotal event that promotes systemic inflammation and elevates the risk of chronic disease development [7]. Mechanistically, pesticides have been shown to induce oxidative stress, cause DNA damage, and dysregulate immune responses, collectively contributing to intestinal inflammation and barrier dysfunction [7,8].

Experimental studies conducted over the past decade consistently show that exposure to herbicides, insecticides, and fungicides can induce overlapping intestinal responses, including oxidative stress, mitochondrial dysfunction, inflammatory activation, epithelial barrier disruption, and increased intestinal permeability. Despite the structural diversity of these compounds, remarkably similar toxicological responses have been observed across pesticide classes, suggesting shared mechanistic pathways underlying intestinal injury [9,10,11,12].

Although numerous experimental studies have examined the intestinal effects of individual pesticides, the available evidence remains fragmented across pesticide classes and experimental models. Previous reviews have addressed specific pesticides, individual pesticide classes, or selected toxicological mechanisms; however, a comprehensive synthesis that compares shared mechanisms of intestinal toxicity across herbicides, insecticides, and fungicides in experimental rodent models remains limited.

This review critically evaluates experimental evidence from rodent models investigating pesticide-induced intestinal toxicity. By integrating findings across herbicides, insecticides, and fungicides, this review identifies the principal mechanistic pathways underlying intestinal injury, with particular emphasis on oxidative stress, inflammatory signalling, epithelial barrier dysfunction, and current knowledge gaps relevant to toxicological research and human health risk assessment.

Because pesticides are widely distributed in environmental matrices and food products, oral exposure is continuous for much of the population. The following section summarizes current evidence on environmental occurrence and human exposure and provides the toxicological context for the mechanistic evidence discussed throughout this review.

## 2. Environmental Occurrence and Human Exposure to Pesticides

The extensive use of pesticides in modern agriculture has led to their widespread presence in environmental compartments, including soil, surface water, groundwater, air, and food products consumed daily by the population [13,14]. Monitoring programs worldwide have consistently detected pesticide residues in fruits, vegetables, cereals, and drinking water, indicating that human exposure is frequent and difficult to avoid [15,16]. Although residue levels are often below established thresholds for acute toxicity, there is growing concern about the potential health consequences of long-term exposure to low doses [3,8].

For the general population, diet is the primary route of exposure. Residues of herbicides, insecticides, and fungicides can persist throughout the food production chain and ultimately reach consumers through contaminated food and beverages [17,18,19,20]. Additional exposure may occur through contaminated water and household dust, contributing to cumulative exposure over time [21,22,23]. Occupational exposure remains a significant concern for agricultural workers, who are repeatedly exposed to concentrated pesticide formulations during handling and application [24], and may result in oral exposure through hand-to-mouth transfer and contamination of food or beverages, representing a potential route for gastrointestinal exposure.

Evidence from human biomonitoring studies further demonstrates the widespread nature of pesticide exposure. Metabolites and parent compounds have been detected in biological samples such as urine, blood, breast milk, and hair, providing direct evidence of continuous contact with these chemicals [25]. However, detecting pesticides or their metabolites in biological samples should be interpreted as evidence of exposure rather than as evidence of intestinal toxicity or adverse health effects. From a toxicological perspective, these findings are particularly relevant because the gastrointestinal tract is an important site of interaction following oral exposure. As a result, intestinal tissues are directly exposed to pesticide residues and their metabolites before systemic distribution occurs [26,27].

Despite increasing recognition of the gut as an important target of environmental toxicants, the extent to which pesticide-induced intestinal alterations contribute to the overall toxicological profile of these compounds remains incompletely characterized. Intestinal endpoints, including epithelial barrier integrity, oxidative stress, inflammatory responses, and alterations in the gut microbiota, have been investigated across experimental models, but their relative contribution to pesticide-induced toxicity and their relevance to human health remain insufficiently defined. Given the central role of the intestinal barrier in maintaining physiological homeostasis, a better understanding of how environmentally relevant pesticide exposures affect intestinal function is essential for refining human health risk assessments and developing more comprehensive toxicological evaluation strategies. Further experimental and translational studies incorporating intestine-specific endpoints may help clarify their relevance for pesticide hazard characterization and human health risk assessment.

## 3. Literature Search Strategy

This study was designed as a critical narrative review aimed at integrating experimental evidence on pesticide-induced intestinal toxicity in rodent models. To support the identification of the relevant literature, searches were conducted in PubMed, Embase, and ScienceDirect for studies published up to March 2026. The search strategy combined terms related to pesticide exposure, intestinal toxicity, and experimental animal models. Representative search combinations included (“pesticide” AND “intestinal damage” AND “animal model”), (“pesticide” AND “intestinal toxicity” AND “mice”), and (“pesticide” AND “intestinal injury” AND “rat”), along with variations incorporating specific pesticide classes and compounds identified during the review process. In Embase, both Emtree terms and free-text keywords were used to maximize search sensitivity. Reference lists of relevant articles were also examined to identify additional studies.

Potentially eligible studies underwent full-text assessment according to predefined inclusion and exclusion criteria. Reference lists of selected articles were also manually reviewed to identify additional relevant studies not retrieved through the electronic database search. Studies were considered relevant if they reported original experimental data from rodent models exposed to herbicides, insecticides, fungicides, or pesticide formulations and evaluated intestinal outcomes such as histopathological alterations, oxidative stress, inflammatory responses, epithelial barrier integrity, permeability, apoptosis, or genotoxicity. Reviews, editorials, conference abstracts, clinical studies, exclusively in vitro investigations, and studies without intestinal endpoints were not included in the mechanistic synthesis.

Because the objective was to provide a critical narrative synthesis rather than a systematic review, study identification and selection were not conducted according to a formal systematic-review protocol, and a PRISMA-based study flow or standardized risk-of-bias assessment was not performed. The included evidence was therefore interpreted qualitatively, with attention to pesticide class, dose, exposure duration, formulation, experimental model, type of intestinal endpoint, consistency across studies, and whether the experimental design supported associative, temporal, or causal interpretations.

## 4. Mechanisms of Pesticide-Induced Toxicity

### 4.1. Oxidative Stress as a Recurrent Mechanism of Intestinal Toxicity

Accumulating evidence identifies oxidative stress as one of the most consistent mechanisms underlying pesticide-induced intestinal toxicity. In rodent models, exposure to herbicides, insecticides, and fungicides disrupts antioxidant defense, promotes lipid peroxidation, and impairs redox homeostasis, ultimately contributing to intestinal dysfunction [12,28,29].

Herbicides with strong redox-cycling capacity, particularly diquat, consistently demonstrate decreased activity of superoxide dismutase and glutathione peroxidase, reduced total antioxidant capacity, elevated malondialdehyde levels, and altered NRF2 signalling in intestinal tissues [30,31,32]. Variability across mouse strains suggests that toxicological responses may depend on dosage and host susceptibility, since DIQ 10 mg/kg b.w. affected GSH-Px activity in ICR mice but did not cause any alteration in this enzyme when 25 mg/kg b.w. was administered to C57BL/6 mice [30,32].

Paraquat, which is known to damage the gastrointestinal, biliary, and nervous systems, in addition to the lungs, kidneys, and liver [33], produces a similarly robust oxidative phenotype characterized by excessive reactive oxygen species generation and suppression of enzymatic antioxidants, often accompanied by inflammatory activation and barrier dysfunction. The inflammatory process, in turn, resulted in intestinal barrier dysfunction, with a consequent decrease in appetite and weight loss, leading to cachexia [34].

Organophosphate insecticides such as chlorpyrifos also promote redox imbalance in intestinal tissue, as shown by decreased glutathione levels, reduced antioxidant enzyme activity, and increased lipid peroxidation [35]. Activation of the KEAP1/NRF2 pathway in some models suggests compensatory antioxidant responses following exposure [35,36].

Among fungicides, pentachlorophenol and thiram exhibit marked oxidative potential, including depletion of sulfhydryl groups, increased protein carbonylation, reduced ferric-reducing capacity, and inhibition of brush border enzymes, indicating that oxidative stress is closely linked to metabolic and structural intestinal injury [9,11,12].

A dose-dependent effect was observed at concentrations ranging from 25 to 150 mg/kg b.w. of pentachlorophenol for both enzymatic and non-enzymatic biomarkers. Collectively, these findings demonstrate that redox imbalance frequently accompanies pesticide-induced intestinal injury across different pesticide classes. However, the available studies do not consistently establish whether oxidative alterations precede inflammatory and epithelial responses or develop concurrently with them.

Although oxidative stress is among the most consistently reported responses across the studies reviewed, its frequent detection should not be interpreted as evidence that it universally represents the initiating event of pesticide-induced intestinal injury. Most studies assessed oxidative biomarkers at a single or limited number of time points and were not specifically designed to establish temporal or causal relationships among redox imbalance, mitochondrial dysfunction, inflammation, and epithelial injury. Accordingly, alterations in ROS production, lipid peroxidation, glutathione status, and antioxidant enzyme activity may represent early toxic responses, downstream consequences of cellular injury, or components of bidirectional interactions with inflammatory and mitochondrial pathways. The available evidence therefore supports oxidative stress as a recurrent and interconnected mechanism of intestinal toxicity, while its precise temporal position within the toxicological sequence appears to depend on the pesticide, exposure conditions, and experimental model.

### 4.2. Inflammatory Activation and Immune Dysregulation

Inflammation is a major axis of pesticide-induced intestinal injury and often occurs alongside oxidative imbalance. Rodent studies consistently report increased pro-inflammatory cytokines, immune cell infiltration, and activation of canonical inflammatory pathways following pesticide exposure [37,38,39,40,41].

Reactive oxygen species function as important upstream mediators by activating NF-κB signalling, which promotes transcription of cytokines such as IL-1, IL-8, and TNF-α and amplifies mucosal immune responses [42]. Herbicides provide consistent evidence of this relationship. Diquat exposure increases NF-κB phosphorylation, macrophage infiltration, and circulating IL-1β, IL-6, and TNF-α, while reducing IL-10 [30]. Paraquat induces time-dependent inflammatory responses characterized by elevated CD11b, iNOS, HMGB1, and IL-1β expression, with early changes detectable within weeks [43]. Acute exposure models show rapid cytokine elevation and increased myeloperoxidase activity [33], and similar oxidative-inflammatory coupling has been described in paraquat-induced lung injury [34]. Glyphosate-based herbicides also exhibit immunomodulatory effects, including increased leukocyte populations, plasma cell differentiation, and lymphocytic inflammation within intestinal tissues [44,45].

Among insecticides, organophosphates such as chlorpyrifos have been associated with intestinal inflammatory responses characterized by necrosis, epithelial edema, cytokine upregulation, increased myeloperoxidase activity, and activation of NF-κB signaling in different experimental models [35,46,47,48]. Intestinal cytokine production has also been proposed as a potential link between local intestinal responses and effects beyond the gastrointestinal tract; however, further evidence is needed to clarify the extent and mechanisms of this relationship [46].

Pyrethroids, including deltamethrin and fenpropathrin, induce inflammatory cell infiltration and time-dependent increases in TNF-α, IL-1β, and IL-6 [47,48]. Other insecticides, such as imidacloprid and monocrotophos, have also been associated with elevated inflammatory markers and intestinal infiltration [49,50].

Fungicides, including epoxiconazole, prothioconazole, mancozeb, and thiram, promote inflammatory responses characterized by immune cell infiltration and increased cytokine expression [9,10,51,52].

Collectively, the studies presented in this review indicate that inflammatory responses are recurrently associated with pesticide-induced intestinal alterations. However, the magnitude and characteristics of these responses vary among pesticide classes, compounds, doses, and exposure conditions. Herbicides, insecticides, and fungicides have all been associated with inflammatory alterations, although the specific mediators and pathways investigated differ considerably across studies. Therefore, the evidence presented here supports inflammation as an important component of pesticide-associated intestinal toxicity, while its causal contribution and relationship with other mechanisms remain incompletely resolved.

### 4.3. Disruption of Epithelial Barrier Integrity

The intestinal epithelium has emerged as a critical yet historically underrecognized target of pesticide toxicity. Increasing evidence shows that chemical exposure is associated with alterations in barrier-related endpoints, including tight junction proteins, mucus production, epithelial turnover, and villus architecture [26,27,53,54,55].

Glyphosate-based herbicides induce dysplasia, leukocyte infiltration, altered adhesion molecule distribution, and changes in tight junction expression after chronic exposure [44,45]. Similarly, prolonged exposure to 2,4-D causes epithelial thinning and dysplastic lesions, suggesting adaptive responses to chronic irritation [56].

Diquat consistently causes severe architectural disruption, including villus disorganization, goblet cell reduction, mucosal swelling, and decreased expression of barrier-related protein such as ZO-1, claudins, and mucins [30,31,32]. Functional assays showing increased serum DAO, D-lactic acid, and FD4 fluorescence further support impaired permeability.

Paraquat produces similar structural deterioration, accompanied by decreased occludin and ZO-1 expression and progressive mucosal damage [33,43].

Organophosphate insecticides have also been associated with barrier-related alterations. Chlorpyrifos reduces tight junction protein expression, increases apoptosis, alters villus morphology, delays intestinal maturation, and elevates circulating permeability markers such as DAO and LPS [35,46,57,58,59].

Other insecticides, including fenpropathrin, imidacloprid, malathion, carbofuran, and monocrotophos, are associated with epithelial detachment, goblet cell depletion, altered crypt architecture, genotoxic events, and increased permeability [47,49,50,60,61].

Fungicides also affect epithelial structure and barrier-related endpoints. Pentachlorophenol inhibits brush border enzymes, damages villi, and induces DNA injury [11,12], while chlorothalonil, folpet, captan, and prothioconazole promote apoptosis, crypt hyperplasia, mucosal degeneration, and architectural distortion [51,62,63,64,65,66]. Epoxiconazole and mancozeb also induce tissue damage and inflammatory infiltration [10,52]. 

Importantly, the evidence for intestinal barrier dysfunction varies according to the endpoints assessed. Changes in the expression or abundance of tight junction proteins, including ZO-1, occludin, and claudins, provide molecular evidence of altered barrier regulation but do not, by themselves, demonstrate increased intestinal permeability. Functional permeability was directly assessed in a smaller subset of studies, whereas circulating markers, such as DAO and LPS, were used as indirect indicators of barrier impairment. These endpoints should therefore be interpreted as complementary, but not equivalent, evidence of intestinal barrier dysfunction. Comparisons among pesticides should also be made cautiously because the reported effects were obtained under heterogeneous exposure conditions, including differences in dose, exposure duration, formulation, and experimental model.

Overall, the available evidence indicates that structural, molecular, and functional barrier-related alterations occur following exposure to several pesticides, although the type and extent of these changes vary according to the compound and experimental conditions. Table 1 summarizes the exposure conditions and corresponding intestinal effects reported in the studies included in this review.

## 5. Mechanistic Convergence Across Pesticide Classes

Despite differences in chemical structure and primary mode of action, recurrent biological responses were identified across the pesticide classes reviewed, particularly alterations in redox homeostasis, inflammatory signaling, and epithelial barrier integrity. However, the available evidence does not support a universal sequence of events applicable to all pesticides. Rather, these processes appear to be interconnected, with their relative contributions and temporal relationships varying according to the compound, dose, exposure duration, formulation, and experimental model (Table 2).

## 6. Body-Weight Alterations Associated with Pesticide Exposure

Alterations in body weight are frequently reported in toxicological studies and may reflect multiple physiological and behavioral responses to pesticide exposure [68]. While some pesticides have been linked to metabolic disruption and obesity [42,69,70], most experimental models examining intestinal toxicity report reduced weight gain or weight loss following exposure.

Herbicides such as diquat and paraquat commonly decrease body weight or food intake, although findings vary depending on exposure duration [30,31,32,33]. In contrast, glyphosate-based herbicides and 2,4-D show minimal effects in some chronic models [45,56].

Organophosphates such as chlorpyrifos frequently reduce body mass across developmental windows, although increases have also been reported following chronic exposure, highlighting the complexity of metabolic responses [35,46,58,59].

Several fungicides, including chlorothalonil, prothioconazole, and folpet, have also been associated with reduced weight gain, whereas others show negligible effects [51,52,63,64].

Rather than representing an isolated endpoint, body weight alterations may reflect multiple physiological responses to pesticide exposure and should therefore be interpreted cautiously. Reduced weight gain or weight loss may result from several factors, including changes in food intake, systemic toxicity, metabolic or endocrine alterations, general health status, and gastrointestinal dysfunction. Damage to the intestinal epithelium may compromise digestive and absorptive capacity through villus atrophy, brush border disruption, reduced activity of digestive enzymes, and alterations in barrier integrity, potentially contributing to changes in body weight. Accordingly, impaired nutrient absorption may be one of several factors contributing to reduced weight gain and metabolic alterations observed in exposed animals. However, most of the studies reviewed were not designed to determine whether intestinal dysfunction directly mediated the observed changes in body weight. Therefore, body weight alterations should be considered a complementary toxicological endpoint rather than a specific indicator or direct consequence of intestinal injury.

Experimental protocols used to induce gastrointestinal damage and the in vivo effects of pesticides on the gastrointestinal tract of rodents are described in detail in Supplementary Tables S1 and S2.

## 7. Integrated Interpretation of Experimental Evidence

This review identifies the intestine as a critical but historically underrecognized target of pesticide toxicity. Across the rodent models reviewed, oxidative imbalance, inflammatory activation, epithelial injury, and barrier dysfunction frequently co-occurred, suggesting interactions among these mechanisms, although their temporal and causal relationships remain incompletely resolved. The direction and temporal sequence of these relationships have not been consistently established and may vary according to pesticide class, dose, exposure duration, formulation, and biological model. The heterogeneity of experimental conditions limits direct comparisons among compounds and prevents broad generalization across pesticides. Most studies evaluated individual pesticides rather than mixtures, limiting conclusions about whether combined exposures produce additive, synergistic, antagonistic, or independent intestinal effects. Studies designed to evaluate pesticide mixtures under environmentally relevant exposure conditions are needed to clarify these potential interactions. Overall, the evidence indicates recurrent intestinal responses across multiple pesticide classes, although responses vary among compounds and experimental conditions. Future studies should use standardized intestinal endpoints, functional measures of barrier integrity, and environmentally relevant exposure scenarios to clarify the mechanistic and translational significance of these findings.

Exposure duration represents an important source of variability among the studies reviewed. Short-term exposure models frequently reported oxidative and inflammatory alterations, whereas studies involving longer exposure periods more often described epithelial, barrier, and histopathological changes. However, these observations derive from different pesticides, doses, formulations, and experimental models and should not be interpreted as evidence of a universal temporal progression of intestinal injury. Table 3 summarizes the intestinal outcomes reported across different exposure-duration categories.

Another source of heterogeneity concerns the use of pure active ingredients versus commercial pesticide formulations. Several experimental studies evaluated formulated products rather than isolated active substances, and the resulting effects cannot necessarily be attributed exclusively to the declared active ingredient. Commercial formulations may contain surfactants, solvents, adjuvants, or other co-formulants capable of modifying local exposure, absorption, and biological responses [71,72]. Direct comparisons between isolated active ingredients and their corresponding formulations were limited among the studies included in this review. Consequently, the present evidence does not clearly distinguish the relative contributions of active ingredients and co-formulants to intestinal toxicity. Future studies should report formulation composition and, where possible, directly compare active ingredients with complete commercial formulations.

## 8. Conclusions and Perspectives

Overall, the evidence synthesized in this review indicates that pesticide exposure can adversely affect gastrointestinal integrity in experimental models, although the magnitude and nature of these effects vary according to the pesticide, exposure conditions, and experimental model. The intestine may represent an important target of pesticide-induced toxicity. Consistent alterations in antioxidant defense systems and inflammatory signaling pathways were observed, suggesting that oxidative imbalance and immune activation may play relevant roles in epithelial injury and may contribute to impaired nutrient absorption, weight loss, or reduced weight gain. Because oral ingestion of contaminated food and water is one of the primary routes of human exposure, understanding the toxicological impact of pesticides on intestinal physiology is critical for improving risk assessment frameworks. Although interspecies differences exist in anatomy, enzymatic activity, and metabolic capacity, rodents share key physiological and molecular characteristics with humans, making them useful experimental models for investigating environmentally induced intestinal toxicity. However, extrapolation of findings from rodents to humans should be interpreted cautiously, given differences in exposure doses, routes of exposure, xenobiotic metabolism, intestinal physiology, and gut microbiota composition. These differences may influence the magnitude and mechanisms of pesticide-induced effects and limit direct translation of experimental findings to human health. 

### 8.1. Strengths and Limitations

This review critically synthesizes experimental evidence from rodent models of pesticide-induced intestinal injury, identifying oxidative stress, inflammatory activation, and epithelial barrier disruption as interconnected mechanisms. The studies reviewed included diverse experimental designs, rodent strains, and pesticide classes, reinforcing the consistency of core mechanistic findings despite methodological differences. However, the exclusive focus on the intestinal epithelium limits the assessment of broader gastrointestinal effects of pesticide exposure, including potential interactions with the gut microbiota.

The limitations identified in the literature include short exposure durations and limited sample sizes in some experimental models, factors that may reduce reproducibility and complicate the interpretation of toxicological outcomes. The lack of studies evaluating pesticide mixtures is also a limiting factor, as it restricts comparisons with real-world exposure scenarios. Nevertheless, the collective body of evidence provides important insights into the intestinal consequences of pesticide exposure. Future research should prioritize well-designed, long-term studies incorporating environmentally relevant exposure scenarios, standardized markers of epithelial integrity, and integrative approaches capable of clarifying the relationship between intestinal injury and systemic disease. Strengthening this evidence base will be essential for improving translational relevance and informing more comprehensive pesticide risk assessment frameworks.

### 8.2. Research Gaps and Future Directions

Although substantial progress has been made in characterizing pesticide-associated intestinal toxicity, important knowledge gaps remain regarding the experimental conditions and endpoints used to investigate these effects. Available studies vary considerably in exposure design, duration, formulation, biological model, and assessment of intestinal outcomes, limiting direct comparisons and broader interpretation of the evidence.

A major gap in the current literature is the limited investigation of pesticide mixtures. This omission is particularly important, as real-world exposures rarely involve single compounds; instead, humans and animals are typically exposed to complex chemical environments in which combined effects may be additive, synergistic, or otherwise unpredictable. Emerging evidence suggests that combined exposures may produce biological effects that cannot be reliably inferred from studies evaluating individual pesticides in isolation. Addressing this gap should therefore be a priority for future toxicological research. Future research should focus on improving the comparability and translational relevance of experimental models while addressing areas that remain insufficiently investigated. These include environmentally relevant exposure scenarios, combined exposures, developmental and sex-specific responses, microbiota interactions, post-exposure recovery, and the identification of intestinal biomarkers applicable across experimental and human studies. The main research gaps identified qualitatively in this narrative review and the corresponding future research needs are summarized in Table 4.

## Figures and Tables

**Table 1:** Experimental exposure conditions and reported intestinal effects of pesticides in rodent models.

Pesticide	Class	Exposure Level(s) Reported	Exposure Duration(s)	Main Intestinal Findings	References
Diquat	Herbicide	10–25 mg/kg; 50 mM	1–3 h; 6 weeks; 1 day; 10 days	Altered antioxidant biomarkers; lipid peroxidation; inflammation; apoptosis; tissue and villus damage; TJ disruption; barrier dysfunction; altered mucus/goblet cells; reduced body weight	[30,31,32,67]
Paraquat	Herbicide	15–30 mg/kg	24 h; 4, 6, and 8 weeks	Increased ROS and lipid peroxidation; altered antioxidant biomarkers and cytokines; TJ disruption; barrier dysfunction; tissue damage; reduced body weight	[33,43]
Glyphosate-based herbicides	Herbicide	25.05–67.54 ppm; 0.075% *w*/*v* in water	Pregnancy to adulthood; 75 days	Inflammation; TJ alterations; altered mucus production; dysplasia	[44,45]
2,4-D	Herbicide	20.69–51.66 ppm	6 months	Dysplasia	[56]
Chlorpyrifos	Insecticide	1–10 mg/kg	30 days; 12 weeks; 1 week; Pregnancy to adulthood	Cytokine alterations; inflammation; tissue damage; TJ disruption; apoptosis; necrosis; altered mucus production; barrier dysfunction; altered antioxidant biomarkers; body-weight alterations	[35,46,57,58,59]
Deltamethrin	Insecticide	0.2 mg/kg/day	8 weeks	Inflammation; apoptosis; tissue damage; reduced body weight	[48]
Carbofuran	Insecticide	2.8–4.0 mg/kg/day	7 and 30 days	DNA damage	[61]
Fenpropathrin	Insecticide	3 mg/kg	1–4 weeks	TJ disruption; lipid peroxidation; decreased SOD; tissue damage; inflammation; increased cytokines	[47]
Imidacloprid	Insecticide	0.06 mg/kg/day	90 days	Increased cytokines; TJ disruption; barrier dysfunction	[49]
Malathion	Insecticide	10 and 50 mg/kg	40 days	Tissue damage; decreased BuChE activity	[60]
Monocrotophos	Insecticide	0.45–1.8 mg/kg/d	30 days	Tissue damage; inflammation	[50]
Captan	Fungicide	6000–16,000 ppm	28 days	Tissue damage	[65,66]
Folpet	Fungicide	6000–16,000 ppm	28 days	Tissue damage, histiocytic cellular infiltrates	[64,65,66]
Pentachlorophenol	Fungicide	25–150 mg/kg	4 and 5 days	Lipid peroxidation; altered antioxidant biomarkers; DNA damage; tissue damage; inflammation	[11,12]
Chlorothalonil	Fungicide	100–300 mg/L	1, 6, and 24 h	Tissue damage; inflammation; apoptosis; increased barrier permeability	[62,63]
Epoxiconazole	Fungicide	0.080 mg/kg/day	6 weeks	Tissue damage; increased barrier permeability; inflammation	[52]
Mancozeb	Fungicide	250 mg/kg	7 weeks	Inflammation; DNA damage; tissue damage	[10]
Prothioconazole	Fungicide	5 mg/kg/day	28 days	Inflammation; tissue damage; altered mucus production; TJ disruption; reduced body weight	[51]
Thiram	Fungicide	100–750 mg/kg	4 days	Altered antioxidant biomarkers; inflammation; tissue damage	[9]

Exposure levels and durations are presented as reported in the original studies and do not necessarily represent matched dose-duration combinations across references. Differences in dose units, routes of administration, exposure durations, pesticide formulations, experimental models, and assessed endpoints limit direct quantitative comparisons across compounds. The findings summarized in this table should therefore not be interpreted as evidence of a generalized dose–response or time-response relationship across pesticide classes.

**Table 2:** Mechanistic convergence across pesticide classes.

Mechanism	Herbicides	Insecticides	Fungicides	Interpretation	Key References
Oxidative stress	Reported	Reported	Reported	Recurrently reported across pesticide classes	[11,32]
NF-κB activation	Reported	Reported	Reported	Evidence of inflammatory signaling, although not consistently assessed across all compounds	[30,35]
Cytokine level alteration	Reported	Reported	Reported	Frequently associated with intestinal inflammatory responses	[33,46,49]
Tight junction disruption	Reported	Reported	Reported	Structural/molecular evidence of barrier alteration; functional permeability was not evaluated in all studies	[30,47,63]
Apoptosis	Reported	Reported	Reported	Observed across pesticide classes, with variability according to compound and exposure conditions	[35,48]
DNA damage	Not identified in reviewed herbicide studies	Reported	Reported	Less consistently investigated than oxidative, inflammatory, and barrier-related endpoints	[12,61]

* “Reported” indicates that the corresponding endpoint was described in studies included in this narrative synthesis and should not be interpreted as a quantitative measure of evidence strength or as evidence of a causal relationship. Differences in experimental design, dose, exposure duration, formulation, and outcome assessment limit direct comparisons across pesticide classes. Abbreviations: ROS, reactive oxygen species; TJ, tight junction; NF-κB, nuclear factor kappa B; ZO, zonula occludens; GPx, glutathione peroxidase; SOD, superoxide dismutase; CAT, catalase; GSH, reduced glutathione; MDA, malondialdehyde; DAO, diamine oxidase; LPS, lipopolysaccharide.

**Table 3:** Intestinal outcomes reported across different pesticide exposure durations.

Exposure Duration	Representative Pesticides	Dominant Findings	References
Acute (≤7 days)	Paraquat, Chlorpyrifos, Carbofuran, Pentachlorophenol, Thiram	Oxidative alterations and cytokine/inflammatory responses	[9,11,12,35,52,61]
Subchronic (1–8 weeks)	Diquat, Paraquat, Fenpropathrin, Deltamethrin, Chlorpyrifos, Malathion, Monocrotophos, Captan and folpet; Epoxiconazole, Mancozeb, Prothioconazole	Barrier alterations, epithelial injury, and apoptosis	[10,43,47,48,50,51,52,60,64,65,66]
Chronic (>8 weeks)	Paraquat, GBH, Chlorpyrifos, 2,4-D, Imidacloprid	Dysplasia, epithelial remodeling	[44,46,49]
Developmental	Chlorpyrifos, GBH	Alterations in intestinal development and maturation	[44,59]

Exposure-duration categories are used descriptively to organize the studies included in this narrative synthesis. Because the studies differed in pesticide, dose, formulation, administration route, experimental model, and assessed endpoints, comparisons across duration categories do not establish a longitudinal or causal sequence of intestinal injury.

**Table 4:** Identified knowledge gaps in pesticide-induced intestinal toxicity.

Research Area	Main Limitation Identified	Future Research Need	Supporting References
Single-compound exposure	Predominance of studies using individual pesticides, frequently under experimental exposure conditions that may not reflect environmentally relevant exposure scenarios	Evaluate intestinal effects under more environmentally relevant exposure conditions and improve comparability across experimental designs	Multiple studies
Mixture toxicity	Limited investigation of combined pesticide exposures in the studies reviewed	Investigate intestinal responses to pesticide mixtures and potential interactions among compounds	-
Chronic low-dose exposure	Limited number of long-term studies using low-dose exposure protocols	Conduct long-term studies using environmentally relevant exposure levels	[46,49]
Developmental exposure	Few studies evaluating intestinal effects across developmental windows and subsequent life stages	Assess persistence and potential long-term consequences of developmental exposure	[59]
Microbiota interactions	Limited mechanistic integration between pesticide-induced intestinal injury and microbiota alterations	Integrate microbiota composition and function with epithelial, inflammatory, and barrier-related endpoints	[46]
Recovery studies	Few experimental designs include post-exposure or withdrawal periods	Determine the reversibility or persistence of intestinal alterations after exposure cessation	[64]
Translational relevance	Limited integration of experimental intestinal endpoints with biomarkers applicable to human studies	Identify and validate comparable biomarkers of intestinal injury and barrier dysfunction across experimental and human studies	Multiple studies

The research gaps summarized in this table were identified qualitatively from the studies included in this narrative review and should not be interpreted as the result of a formal evidence-grading or research-priority assessment.

## Data Availability

No new data were created or analyzed in this study. Data sharing is not applicable to this article.

## Supplementary Materials

The following supporting information can be downloaded at: https://www.mdpi.com/article/10.3390/cimb48101083/s1.
