# Supplementary material for: Mechanistic Pathways of Pesticide-Induced Intestinal Injury: Oxidative Stress, Inflammation, and Barrier Dysfunction
Source: Curr Issues Mol Biol. 2026 Oct 20;48(10):1083. doi: 10.3390/cimb48101083 (PMC13702966; doi:10.3390/cimb48101083)
Supplement: Supplementary file 1 [file cimb-48-01083-s001.zip › cimb-4542694-supplementary.pdf]

Mechanistic Pathways of Pesticide-Induced Intestinal Injury: Oxidative Stress, Inflammation, and Barrier Dysfunction

Nathalia Kirsten [1,2], Shayane Milhorini da Silva [1,2], Camila Bach de Assis [1,2], Salvador Paganella Chaves Júnior [1,2], Izonete Cristina Guiloski [1,2] and Daniele Maria-Ferreira [1,2,*]

[1] Faculdades Pequeno Príncipe, Av. Iguaçu, 333, Rebouças, Curitiba CEP 80230-020, PR, Brazil
[2] Instituto de Pesquisa Pelé Pequeno Príncipe, Av. Silva Jardim, 1632, Água Verde, Curitiba CEP 80250-060, PR, Brazil
* Correspondence: danielemariaferreira@gmail.com

Supplementary Material

Supplementary Table S1. Experimental protocols used to induce gastrointestinal damage

Pesticide	Animal model	Experimental groups	Intestinal tissue	Reference
Herbicide<br>Diquat	Male Sprague Dawley CD rats	Experimental groups for 1, 2, and 3 hours<br>Group 1: Control<br>Group 2: Diquat (10 mM)<br>Group 3: Diquat (50 mM)<br>Group 4: Diquat (100 mM)<br><br>n = 4-5<br><br>Directly into the lumen of the jejunum	Jejunum	[1]
Herbicide<br>Diquat	Male C57BL/6 mice	Group 1: Control<br>Group 2: Diquat (25 mg/kg bw)<br><br>n =10<br><br>Intraperitoneal for 1 day	Jejunum	[2]
Herbicide<br>Diquat	Male C57BL/6 mice	Group 1: Control<br>Group 2: Diquat (25 mg/kg)<br><br>12 hours after the intraperitoneal injection	Jejunum	[3]

Herbicide<br>Diquat	Male ICR mice	Group 1: control<br>Group 2: Diquat (10 mg/kg - 2x)<br><br>n =10<br><br>10 days of experiment; Intraperitoneal injection on day 1 and day 4	Duodenum, jejunum, and colon	[4]
Herbicide<br>Paraquat	Male C57BL/6 mice	Group 1: Control<br>Group 2: Paraquat (30 mg/kg)<br><br>n = 8<br><br>Intraperitoneal for 24 hours	Jejunum	[5]
Herbicide<br>Paraquat	Male C57BL/6J mice	4 weeks exposure<br>Group 1: Control<br>Group 2: Paraquat (15 mg/kg)<br><br>6 weeks exposure<br>Group 1: Control<br>Group 2: Paraquat (15 mg/kg)	Colon	[6]

		8 weeks exposure<br>Group 1: Control<br>Group 2: Paraquat (15 mg/kg)<br><br>n = 10<br><br>Intraperitoneal for 4, 6 and 8 weeks		
Herbicide<br>Glyphosate-based herbicide (GBH) (Roundup)	Male and Female Wistar rats	Inhalation exposure<br>Group 1: Control<br>Group 2: GBH (27.05 ppm)<br>Group 3: GBH (45.27 ppm)<br>Group 4: GBH (67.54 ppm)<br><br>Oral exposure - nebulized feed<br>Group 1: Control<br>Group 2: GBH (27.05 ppm)<br>Group 3: GBH (45.27 ppm)<br>Group 4: GBH (67.54 ppm)<br>Positive control - bone marrow micronucleus test<br>Cyclophosphamide (50 mg/kg - single dose)	Small and large intestine	[7]

		n = 10 for exposed and negative control; n = 8 for positive control for the bone marrow micronucleus test<br><br>Inhalation and oral (nebulized feed) for 75 days		
Herbicide<br>Glyphosate-based herbicide (GBH) (Roundup)	Male and Female Swiss mice	5 groups:<br>Group 1: Control<br>Group 2: Pups exposed to GBH (0.075% w/v in water) for 30 days<br>Group 3: Pups exposed to GBH (0.075% w/v in water) for 60 days<br><br>n = NI<br><br>Via drinking water during pregnancy until adulthood (pregnancy, through lactation, weaning, and adulthood)	Colon	[8]
Herbicide<br>2,4-	Male Wistar rats	Inhalation exposure<br>Group 1: Control<br>Group 2: 2,4-D (20.69 ppm)	Jejunum, ileum, and colon	[9]

Dichlorophenoxyacetic acid (2,4-D)		Group 3: 2,4-D (34.63 ppm)<br>Group 4: 2,4-D (51.66 ppm)<br><br>Oral exposure - nebulized feed<br>Group 1: Control<br>Group 2: 2,4-D (20.69 ppm)<br>Group 3: 2,4-D (34.63 ppm)<br>Group 4: 2,4-D (51.66 ppm)<br><br>n =10<br><br>Inhalation (nebulization 5 days/week) and oral (nebulized feed every 2 days) for 6 months		
Insecticide Chlorpyrifos (CPF)	Female (pregnant) and male (pups) Hannover Wistar rats	Day 21 (weaning)<br>Group 1: Control (CPF0) (n =10)<br>Group 2: CPF1 (1mg/kg) (n =10)<br>Group 3: CPF5 (5 mg/kg) (n =8)<br><br>Day 60 (adulthood)<br>Group 1: Control (CPF0) (n =10)<br>Group 2: CPF1 (1mg/kg) (n =10)	Ileum, cecum, and colon	[10]

		Group 3: CPF5 (5 mg/kg) (n =9)<br><br>Pregnant rats were gavaged. Pups were exposed in utero and lactational (for Day 21) or after further exposure to CPF by gavage (for 60 days)		
Insecticide Chlorpyrifos (CPF)	Male Mus musculus KM mice	Group 1: Control<br>Group 2: CPF (1 mg/kg bw)<br><br>n =5<br><br>Gavage for 30 days	Colon	11
Insecticide Chlorpyrifos (CPF)	Male C57BL/6 and ICR mice	Group 1: Control (normal fat diet)<br>Group 2: CPF (5 mg/kg; normal fat diet)<br><br>n = 8<br><br>Gavage for 12 weeks	Ileum and colon	12
Insecticide Chlorpyrifos (CPF)	Female SD rats	Group 1: Control<br>Group 2: CPF (10 mg/kg bw/day)<br>n = 8	Duodenum, jejunum, and ileum, liver,	13

		Gavage for 1 week	spleen, and kidney	
Insecticide Chlorpyrifos (CPF)	Male C57BL/6 mice	Group 1: Control<br>Group 2: CPF (5 mg/kg)<br><br>n = 8<br><br>Gavage for 1 week	Small intestine	[14]
Insecticide Deltamethrin	Female BALB/c mice	Group 1: Control<br>Group 2: Deltamethrin (0.2 mg/kg/day<br><br>n =15<br><br>Gavage for 8 weeks	Colon	[15]
Insecticide Carbofuran	Male Wistar rats	7 days<br>Group 1: Control<br>Group 2: Carbofuran (4 mg/kg bw)<br><br>30 days<br>Group 1: Control<br>Group 2: Carbofuran (2.8 mg/kg bw)	Isolated cells from the small intestine up to cecum	[16]

		n = 6–8<br><br>Orally using Ryle's tube		
Insecticide Fenpropathrin	Male SD rats	Transport of gliquidone on the using chamber system (n = 3):<br>Group 1: control<br>Group 2: Fenpropathrin (3 mg/kg) - 2 weeks<br><br>Other assays (n = 5):<br>Group 1: control<br>Group 2: Fenpropathrin (3 mg/kg) - 1 week<br>Group 3: Fenpropathrin (3 mg/kg) - 2 weeks<br>Group 4: Fenpropathrin (3 mg/kg) - 4 weeks<br><br>Gavage<br>Transport of gliquidone on the using chamber system: 2 weeks<br>Other assays: 1; 2; 3; and 4 weeks	Jejunum	[17]
Insecticide Imidacloprid	Male Wistar rats	Group 1: control<br>Group 2: Imidacloprid (0.06 mg/kg body	Colon	[18]

		weight/day)<br><br>n =10<br><br>Gavage for 90 days		
Insecticide Malathion (MAL)	Male Wistar rats	Group 1: control<br>Group 2: MAL 10 mg/kg<br>Group 3: MAL 50 mg/kg<br><br>First stage n = 5-16 (macroscopic and histological analyses, evaluation of the enzyme BuChE, and for immunofluorescence techniques for the evaluation of ENS);<br>Second stage n = 10-17 (faecal pellets phenotype and intestinal motility<br><br>Orally for 40 days	Colon	[19]
Insecticide Monocrotophos (MCP)	Male Wistar rats	4 groups (n = 6):<br>Group 1: control<br>Group 2: MCP (0.45 mg/kg b.w/d)<br>Group 3: MCP (0.9 mg/kg b.w/d)	Jejunum<br>*for the motility assay it was used	[20]

		Group 4: MCP (1.8 mg/kg b.w/d)<br><br>n = 6 each group<br><br>Via drinking water for 30 days	the whole small intestine	
Fungicides Captan and Folpet	Female B6C3F1 mice	Group 1: Control<br>Group 2: Captan (6,000 ppm)<br>Group 3: Captan (16,000/12,000 ppm)*<br>Group 4: Folpet (6,000 ppm)<br>Group 5: Folpet (16,000 ppm)<br><br>n = 20 each group, which 10 were allowed to recover for 28 days<br><br>The animals were exposed to fungicides for 28 days through the feed.<br>*Day 10: the dose concentration was decreased from 16,000 to 12,000 ppm	Duodenum	[21]
Fungicides Captan and Folpet	Female B6C3F1 mice	Group 1: Control<br>Group 2: Captan (6,000 ppm)<br>Group 3: Captan (16,000/12,000 ppm)*	Duodenum	[22]

		Group 4: Folpet (6,000 ppm)<br>Group 5: Folpet (16,000 ppm)<br><br>n = 20 each group, which 10 were allowed to recover for 28 days<br><br>The animals were exposed to fungicides for 28 days through the feed.<br>*Day 10: the dose concentration was decreased from 16,000 to 12,000 ppm		
Fungicide<br>Folpet	Male and Female ICR mice	Group 1: Control<br>Group 2: Folpet (6000 ppm)<br><br>n = 6<br><br>The animals were exposed to fungicide for 7, 14, or 28 days (six mice each), and an additional 12 mice were administered the test diet for 28 days and then fed the control diet for 17 days.	Duodenum and cecum	[23]

Fungicide Pentachlorophenol (PCP)	Male albino Wistar rats	Group 1: Control<br>Group 2: PCP (25 mg/kg bw)<br>Group 3: PCP (50 mg/kg bw)<br>Group 4: PCP (100 mg/kg bw)<br>Group 5: PCP (150 mg/kg bw)<br><br>n = 5<br><br>Gavage for 5 days	Intestine (entire lenght) and duodenum for histopathology	24
Fungicide Pentachlorophenol (PCP)	Male Wistar rats	Group 1: Control<br>Group 2: PCP (125 mg/kg bw/day)<br><br>n =5<br><br>Orally for 4 days	Intestine (entire lenght) and duodenum for histopathology	25
Fungicide Chlorothalonil (CTL)	Male Sprague Dawley rats	Group 1: Control<br>Group 2: CTL (200 mg/kg 1 hour)<br>Group 3: CTL (2000 mg/kg 1 hour)<br>Group 4: CTL (200 mg/kg 6 hours)<br>Group 5: CTL (2000 mg/kg 6 hours)<br>Group 6: CTL (200 mg/kg 24 hours)	Distal intestine	26

		Group 7: CTL (2000 mg/kg 24 hours)<br><br>n = 1 (3 independent experiments; 1 rat per dose were performed for each sampling time)<br><br>Orally (in corn oil)		
Fungicide Chlorothalonil (CTL)	Male ICR mice	Group 1: Control<br>Group 2: CTL (100 mg/L)<br>Group 3: CTL (300 mg/L)<br><br>n = 8<br><br>Intragastric for 7 days	Colon and ileum	[27]
Fungicide Epoxiconazole	Male ICR mice	Group 1: Control<br>Group 2: Epoxiconazole (0.120 mg/kg)<br><br>n= 6<br><br>Gavage for 6 weeks	Colon	[28]
Fungicide Mancozeb	Male Sprague Dawley rats	Group 1: Control (1 ml/100 g corn oil)<br>Group 2: Mancozeb (250 mg/kg, twice a week for	Colon	[29]

		7 weeks)<br>Group 3: Mancozeb (250 mg/kg, twice a week for 7 weeks, then treatment was interrupted for 5 weeks)<br><br>n =10<br><br>Gavage		
Fungicide<br>Prothioconazole and Prothioconazole-desthio	Male ICR mice	Group 1: Control<br>Group 2: Prothioconazole (5 mg/kg bw/day)<br>Group 3: Prothioconazole-desthio (5 mg/kg bw/day)<br><br>n = 8<br><br>Gavage for 28 days	Colon, liver	[30]
Fungicide<br>Thiram	Male Sprague Dawley rats	Group 1: Control<br>Group 2: Thiram (100 mg/kg)<br>Group 3: Thiram (250 mg/kg)<br>Group 4: Thiram (500 mg/kg)<br>Group 5: Thiram (750 mg/kg)	Small intestine and small intestinal mucosa (for the preparation of	[31]

		n = NI<br><br>The animals were exposed to fungicide by oral gavage once a day for 4 days	brush border membranes, brush border membrane vesicles and homogenates)	

*Only exhibiting results of pesticides; NI = not informed.

ICR mice (**Institute** of Cancer Research mice)

Supplementary Table S2. In vivo effects of pesticides on the gastrointestinal tract of rodents.

Pesticide	Antioxidant system	Inflammation	Intestinal damage and permeability	Body weight and food intake	Reference
Herbicide Diquat	NE	NE	Enterocytic exfoliation, distortion of the intestinal villi<br><br>↑ ratio of goblet cells to enterocytes at the tips of the villi	NE	[1]
Herbicide Diquat	↓ jejunal and serum SOD<br><br>↑ T-AOC, MDA	NE	↓ of the mRNA of MUC-2 and a down-regulation in the protein expression of occludin and claudin-1 in the jejunum<br><br>↑ DAO and D-LA, villus height, crypt depth, goblet cells<br><br>disarranged jejunal villi, swelling in the intestinal mucosa	↓ body weight	[2]
Herbicide Diquat	↓ SOD jejunal activity	NE	↑ DAO and D-LA	No statistical difference	[3]

	↑ jejunal and serum MDA		discontinuous brush borders and blunt villi in the jejunum, intestinal permeability		
Herbicide Diquat	↓ SOD, GPx, T-AOC, and Nrf2 expression<br><br>↑ MDA	↑ IL-1β, IL-6, TNF-α<br>↓ IL-10<br><br>↑ NF-κB (p65) expression and macrophage infiltration	↑ DAO and D-LA<br><br>scattered intestinal villi, sloughing and shortening of intestinal epithelium in the jejunum and colon, and pathological swelling in the submucosa of the duodenum<br><br>↓ ZO-1, ZO-2, Claudin-1, Mucin-1, Mucin-2, and Occludin<br><br>apoptosis	↓ avarege weight	[4]
Herbicide Paraquat	↓ SOD, catalase, and GPx<br><br>↑ ROS in jejunal tissue, MDA level	↑ IL-1β, IL-6, TNF-α, IFN-y, MPO	↓ ratio of villus height to crypt depth, goblet cells number, in the thickness of the mucosal epithelium, and deteriorated structure of jejunal villi	↓ body weight and food intake	[5]

			↓ D-LA and DAO		
Herbicide<br>Paraquat	NE	↑ IL-1β, CD11b, CD11b, iNOS, HMGB1	damage to the tissue<br>dysfunction of the gut<br><br>↓ ZO-1 and Occludin	NE	[6]
Herbicide<br>Glyphosate-based herbicide (GBH) (Roundup)	NE	lymphocyte infiltration and tissue congestion	↑ NORs in the tongue mucosa<br>dysplasia of the epithelium and karyomegaly	No statistical difference	[7]
Herbicide<br>Glyphosate-based herbicide (GBH) (Roundup)	NE	increased infiltration of leukocytes in colonic lamina propria<br><br>↑ Arginase-1<br><br>↓ Iba-1<br>presence of CD8- and CD-138-positive	30-day-old mice: ↓ ZO-2<br>60-day-old mice: ↑ ZO-2<br><br>change in the location of expression of syndecan-1	NE	[8]

		lymphocytes and plasma cells<br><br>differentiation of B lymphocytes into plasma cells in the colon			
Herbicide 2,4-Dichlorophenoxyacetic acid (2,4-D)	NE	NE	hyperkeratosis in the esophagus, thinning of the epithelial thickness, nucleoli-organizing regions (NORs), mild dysplasia	No statistical difference	[9]
Insecticide Chlorpyrifos	NE	NE	↓ villus height and width, crypt depth, epithelial thickness, thickness of both the circular and longitudinal muscle layers in the exposed group<br><br>↑ expression of Muc2 mRNA	↓ body mass and lenght	[10]
Insecticide Chlorpyrifos	NE	intestinal epithelial edema	necrosis; ↑ DAO and LPS	NE	[11]

Insecticide Chlorpyrifos	NE	↑ IL-1 β, TNF-α, MCP-1, PAI-1 expression	↓ mRNA expression of occludin, claudin 1, and ZO-1<br><br>intestinal permeability	↑ weight gain	12
Insecticide Chlorpyrifos	↑ Nrf2 expression in the duodenum and jejunum, upregulation of GPx1 gene expression in jejunum	↑ IL-1β, IL-6, TNF-α, NF-Nβ mRNA expression	disarrangement and damage to the villi in the duodenum and jejunum<br><br>↓ in villus height (jejunum)<br>↑ crypt depth (duodenum)<br><br>↓ ZO-1, Claudin-1, and Occludin<br><br>↑ apoptotic cells<br>upregulation of pro-apoptotic genes	↓ body weight	13
Insecticide Chlorpyrifos	↑ serum ROS, MDA in intestinal tissue<br>↓T-AOC, GSH, SOD, CAT, and GPx in intestinal tissue	↑ IL-1β, TNF-α<br>↑ p-IKKα/β and p-IκBα expression	↑ DAO<br>↑ apoptotic cells<br><br>shedding of intestinal villi, cell necrosis, and alterations in tissue density	↓ body weight	14

			↓ ZO-1 and Occludin		
Insecticide<br>Deltamethrin	NE	infiltration of<br>inflammatory cells	↑ apoptotic cells<br><br>shortened colon length<br>tissue damage	↓ body weight	15
Insecticide<br>Carbofuran	NE	NE	↑ micronucleus in enterocytes	NE	16
Insecticide<br>Fenpropathrin	↓ SOD<br>↑ MDA	↑ IL-1β, IL-6 and<br>TNF-α, expression	↓ goblet cells, Claudin-1, ZO-1<br>and occludin<br><br>damage at the tip of the intestinal<br>villi, and separation of the lamina<br>propria from the cells<br>intestinal permeability	NE	17
Insecticide<br>Imidacloprid	NE	↑ IL-1β, TNF-α	↓ Claudin-1, Occludin, and ZO-1<br>↑ endotoxin in the form of<br>lipopolysaccharide<br><br>intestinal permeability	No statistical<br>difference	18

Insecticide<br>Malathion (MAL)	NE	NE	atrophy of the mucosa (lowest dose), and hypertrophy<br>↑ depth of the crypts (highest dose)<br>↓ goblet cells and BuChE activity	NE	[19]
Insecticide<br>Monocrotophos (MCP)	NE	infiltration of inflammatory cells	altered villi morphology<br>necrosis<br>↑ goblet cells<br><br>inhibition of BuChE activity in the intestine<br>higher rate of intestinal transit<br><br>alteration of the fluidity in the membrane function	NE	[20]
Fungicides<br>Captan and Folpet	NE	NE	crypt cell hyperplasia, villous hypertrophy, ↑ villous enterocytes, histiocytic cells in the villus lamina propria, ↑ mononuclear cell infiltrates in villi, rarefaction	No statistical difference	[21]

			of the lamina propria, thickened villi		
Fungicides Captan and Folpet	↑ Nox2	NE	alteration of pathways related to histological events (cell proliferation, growth, and signaling)	NE	[22]
Fungicide Folpet	NE	infiltration of lymphocytes in the lamina propria	distended cecum, thickening of the duodenum, crypt cell hyperplasia and, villous hypertrophy	↓ body weight	[23]
Fungicide Pentachlorophenol	↓GSH, total-SH, LPO, PO, antioxidant capacity, SOD, GPx, and TR<br><br>↑ H2O2, CAT and GST	NE	↓ BBM and carbohydrate metabolism enzymes<br><br>gap between epithelium and lamina propria, shedding of epithelial lining, damage to epithelial cells and villi; DNA damage	NE	[24]
Fungicide Pentachlorophenol	↓ ability to reduce ferric ions and neutralize free	infiltration of inflammatory cells in lamina propria	↓ BBM and carbohydrate metabolism enzymes	NE	[25]

	radicals, total-SH, GSH, SOD, GPx, and GSR<br><br>↑ free radicals, MDA and carbonyl protein content		swelling of the villi and loss of the epithelial lining over the villi, damage to the crypts and lamina propria infiltration<br><br>DNA damage		
Fungicide Chlorothalonil	NE	NE	NE	NE	26
Fungicide Chlorothalonil	NE	NE	↓ of MUC2<br>↑ apoptotic cells<br>intestinal permeability<br><br>villi degeneration, hypotrophy and hypoplasia of goblet cells with a decreased number and size	↓ body weight	27
Fungicide Epoxiconazole	NE	infiltration of inflammatory cells	↓ mucosal width and villus length, muscle layer width<br><br>infiltration of intestinal inflammatory cells	No statistical difference	28

			↑ DAO and LPS		
Fungicide Mancozeb	NE	congestion and infiltration of inflammatory cells in lamina propria and submucosa	DNA damage in the colon<br><br>desquamation of epithelium and mild congestion	NE	29
Fungicide Prothioconazole and Prothioconazole-desthio	NE	infiltration of inflammatory cells	ulceration, dysplasia, and architectural distortion<br><br>↑ TFF3<br>↓ Occludin, MUC2, and MUC3	↓ body weight gain	30
Fungicide Thiram	↓ CAT, GSH and total SH, GSR, TR, GPx, GST and antioxidant capacity<br><br>↑ SOD, protein carbonyl content, H_2O_2	congestion of lamina propria of villi<br>↑ lymphocytic infiltration	↓ BBM enzymes<br><br>vacuolation and degeneration of enterocytes and partial loss of the apical part of the villi	NE	31

*NE = not evaluated

References

1. Rawlings, J.M.; Foster, J.R.; Heylings, J.R. Diquat-Induced Intestinal Secretion in the Anaesthetized Rat. *Hum. Exp. Toxicol.* **1992**, *11*, 524–529. https://doi.org/10.1177/096032719201100614.
2. Qiao, L.; Dou, X.; Yan, S.; Zhang, B.; Xu, C. Biogenic selenium nanoparticles synthesized by *Lactobacillus casei* ATCC 393 alleviate diquat-induced intestinal barrier dysfunction in C57BL/6 mice through their antioxidant activity. *Food Funct.* **2020**, *11*, 3020–3031. https://doi.org/10.1039/d0fo00132e.
3. Hao, L.; Cheng, Y.; Su, W.; Wang, C.; Lu, Z.; Jin, M.; Wang, F.; Wang, Y. Pediococcus pentosaceus ZJUAF-4 relieves oxidative stress and restores the gut microbiota in diquat-induced intestinal injury. *Appl. Microbiol. Biotechnol.* **2021**, *105*, 1657–1668. https://doi.org/10.1007/s00253-021-11111-6.
4. Zhang, H.; Liu, Y.; Fang, X.; Gu, L.; Luo, C.; Chen, L.; Wang, Q. Vitamin D_3 Protects Mice from Diquat-Induced Oxidative Stress through the NF-*κ*B/Nrf2/HO-1 Signaling Pathway. *Oxidative Med. Cell. Longev.* **2021**, *2021*, 6776956. https://doi.org/10.1155/2021/6776956.
5. Qi, M.; Liao, S.; Wang, J.; Deng, Y.; Zha, A.; Shao, Y.; Cui, Z.; Song, T.; Tang, Y.; Tan, B.; et al. MyD88 deficiency ameliorates weight loss caused by intestinal oxidative injury in an autophagy-dependent mechanism. *J. Cachexia Sarcopenia Muscle* **2022**, *13*, 677–695. https://doi.org/10.1002/jcsm.12858.
6. Wang, K.; Zhang, C.; Zhang, B.; Li, G.; Shi, G.; Cai, Q.; Huang, M. Gut dysfunction may be the source of pathological aggregation of alpha-synuclein in the central nervous system through Paraquat exposure in mice. *Ecotoxicol. Environ. Saf.* **2022**, *246*, 114152. https://doi.org/10.1016/j.ecoenv.2022.114152.
7. Serra, F.d.M.; Parizi, J.L.S.; Odorizzi, G.A.S.d.M.; Sato, G.M.R.H.; Patrão, I.B.; Chagas, P.H.N.; Mello, F.d.A.; Nai, G.A. Subchronic exposure to a glyphosate-based herbicide causes dysplasia in the digestive tract of Wistar rats. *Environ. Sci. Pollut. Res.* **2021**, *28*, 61477–61496. https://doi.org/10.1007/s11356-021-15051-6.
8. Del Castilo, I.; Neumann, A.S.; Lemos, F.S.; De Bastiani, M.A.; Oliveira, F.L.; Zimmer, E.R.; Rêgo, A.M.; Hardoim, C.C.P.; Antunes, L.C.M.; Lara, F.A.; et al. Lifelong Exposure to a Low-Dose of the Glyphosate-Based Herbicide RoundUp® Causes Intestinal Damage, Gut Dysbiosis, and Behavioral Changes in Mice. *Int. J. Mol. Sci.* **2022**, *23*, 5583. https://doi.org/10.3390/ijms23105583.
9. Mariotti, V.; Naufal, I.; Amorim, I.; Parizi, J.; Nai, G. Digestive tract toxicity associated with exposure to 2,4-dichlorophenoxyacetic acid in rats. *Braz. J. Med. Biol. Res.* **2022**, *55*, e12350. https://doi.org/10.1590/1414-431x2022e12350.
10. Condette, C.J.; Bach, V.; Mayeur, C.; Gay-Quéheillard, J.; Khorsi-Cauet, H. Chlorpyrifos Exposure During Perinatal Period Affects Intestinal Microbiota Associated with Delay of Maturation of Digestive Tract in Rats. *J. Pediatr. Gastroenterol. Nutr.* **2015**, *61*, 30–40. https://doi.org/10.1097/mpg.0000000000000734.
11. Zhao, Y.; Zhang, Y.; Wang, G.; Han, R.; Xie, X. Effects of chlorpyrifos on the gut microbiome and urine metabolome in mouse (Mus musculus). *Chemosphere* **2016**, *153*, 287–293. https://doi.org/10.1016/j.chemosphere.2016.03.055.
12. Liang, Y.; Zhan, J.; Liu, D.; Luo, M.; Han, J.; Liu, X.; Liu, C.; Cheng, Z.; Zhou, Z.; Wang, P. Organophosphorus pesticide chlorpyrifos intake promotes obesity and insulin resistance through impacting gut and gut microbiota. *Microbiome* **2019**, *7*, 19. https://doi.org/10.1186/s40168-019-0635-4.
13. Fu, H.; Liu, H.; Ge, Y.; Chen, Y.; Tan, P.; Bai, J.; Dai, Z.; Yang, Y.; Wu, Z. Chitosan oligosaccharide alleviates and removes the toxicological effects of organophosphorus pesticide chlorpyrifos residues. *J. Hazard. Mater.* **2022**, *446*, 130669. https://doi.org/10.1016/j.jhazmat.2022.130669.
14. Liu, S.; Zhu, X.; Pei, H.; Zhao, Y.; Zong, Y.; Chen, W.; He, Z.; Du, R. Ginseng Stem-and-Leaf Saponins Mitigate Chlorpyrifos-Evoked Intestinal Toxicity In Vivo and In Vitro: Oxidative Stress, Inflammatory Response and Apoptosis. *Int. J. Mol. Sci.* **2023**, *24*, 15968. https://doi.org/10.3390/ijms242115968.
15. Ma, R.; Sun, T.; Wang, X.; Ren, K.; Min, T.; Xie, X.; Wang, D.; Li, K.; Zhang, Y.; Zhu, K.; et al. Chronic exposure to low-dose deltamethrin can lead to colon tissue injury through PRDX1 inactivation-induced mitochondrial oxidative stress injury and gut microbial dysbiosis. *Ecotoxicol. Environ. Saf.* **2023**, *264*, 115475. https://doi.org/10.1016/j.ecoenv.2023.115475.
16. Gera, N.; Kiran, R.; Mahmood, A. Carbofuran administration induces genotoxic effects in epithelial cells across crypt–villus axis in rat intestine. *Pestic. Biochem. Physiol.* **2011**, *100*, 280–283. https://doi.org/10.1016/j.pestbp.2011.04.013.

17. Xu, L.; Shen, W.; Liu, Y.; Zhang, M.; Yang, Y.; Yin, D. Fenpropathrin increases gliquidone absorption via causing damage to the integrity of intestinal barrier. *Ecotoxicol. Environ. Saf.* **2022**, *242*, 113882. https://doi.org/10.1016/j.ecoenv.2022.113882.
18. Zhao, G.-P.; Wang, X.-Y.; Li, J.-W.; Wang, R.; Ren, F.-Z.; Pang, G.-F.; Li, Y.-X. Imidacloprid increases intestinal permeability by disrupting tight junctions. *Ecotoxicol. Environ. Saf.* **2021**, *222*, 112476. https://doi.org/10.1016/j.ecoenv.2021.112476.
19. Pupim, A.; Basso, C.; Machado, C.; Watanabe, P.; Fernandes, G.; ErthalL, R.; Sodré, G.; Guarnier, F.; Simão, A.; Araújo, E. Long-term and low dose oral malathion exposure causes morphophysiological changes in the colon of rats. *Life Sci.* **2023**, *327*, 121840. https://doi.org/10.1016/j.lfs.2023.121840.
20. Vismaya; Rajini, P. Oral exposure to the organophosphorus insecticide, Monocrotophos induces intestinal dysfunction in rats. *Food Chem. Toxicol.* **2014**, *71*, 236–243. https://doi.org/10.1016/j.fct.2014.05.030.
21. Thompson, C.M.; Wolf, J.C.; McCoy, A.; Suh, M.; Proctor, D.M.; Kirman, C.R.; Haws, L.C.; Harris, M.A. Comparison of Toxicity and Recovery in the Duodenum of B6C3F1 Mice Following Treatment with Intestinal Carcinogens Captan, Folpet, and Hexavalent Chromium. *Toxicol. Pathol.* **2017**, *45*, 1091–1101. https://doi.org/10.1177/0192623317742324.
22. Chappell, G.A.; Rager, J.E.; Wolf, J.; Babic, M.; LeBlanc, K.J.; Ring, C.L.; Harris, M.A.; Thompson, C.M. Comparison of Gene Expression Responses in the Small Intestine of Mice Following Exposure to 3 Carcinogens Using the S1500+ Gene Set Informs a Potential Common Adverse Outcome Pathway. *Toxicol. Pathol.* **2019**, *47*, 851–864. https://doi.org/10.1177/0192623319873882.
23. Gordon, E.; Cohen, S.M.; Singh, P. Folpet-induced short term cytotoxic and proliferative changes in the mouse duodenum. *Toxicol. Mech. Methods* **2011**, *22*, 54–59. https://doi.org/10.3109/15376516.2011.593054.
24. Maheshwari, N.; Khan, A.A.; Ali, A.; Mahmood, R. Oral administration of pentachlorophenol impairs antioxidant system, inhibits enzymes of brush border membrane, causes DNA damage and histological changes in rat intestine. *Toxicol. Res.* **2022**, *11*, 616–627. https://doi.org/10.1093/toxres/tfac035.
25. Maheshwari, N.; Khan, A.A.; Mahmood, R. Oral administration of curcumin and gallic acid alleviates pentachlorophenol-induced oxidative damage in rat intestine. *Food Chem. Toxicol.* **2023**, *176*, 113745. https://doi.org/10.1016/j.fct.2023.113745.
26. Godard, T.; Fessard, V.; Huet, S.; Mourot, A.; Deslandes, E.; Pottier, D.; Hyrien, O.; Sichel, F.; Gauduchon, P.; Poul, J.-M. Comparative in vitro and in vivo assessment of genotoxic effects of etoposide and chlorothalonil by the comet assay. *Mutat. Res. Toxicol. Environ. Mutagen.* **1999**, *444*, 103–116. https://doi.org/10.1016/s1383-5718(99)00100-x.
27. Tao, H.; Wang, J.; Bao, Z.; Jin, Y.; Xiao, Y. Acute chlorothalonil exposure had the potential to influence the intestinal barrier function and micro-environment in mice. *Sci. Total. Environ.* **2023**, *894*, 165038. https://doi.org/10.1016/j.scitotenv.2023.165038.
28. Sun, W.; Yan, S.; Meng, Z.; Tian, S.; Jia, M.; Huang, S.; Wang, Y.; Zhou, Z.; Diao, J.; Zhu, W. Combined ingestion of polystyrene microplastics and epoxiconazole increases health risk to mice: Based on their synergistic bioaccumulation in vivo. *Environ. Int.* **2022**, *166*, 107391. https://doi.org/10.1016/j.envint.2022.107391.
29. Yahia, D.; O El-Amir, Y.; Rushdi, M. Mancozeb fungicide-induced genotoxic effects, metabolic alterations, and histological changes in the colon and liver of Sprague Dawley rats. *Toxicol. Ind. Health* **2019**, *35*, 265–276. https://doi.org/10.1177/0748233719834150.
30. Meng, Z.; Huang, S.; Sun, W.; Yan, S.; Chen, X.; Diao, J.; Zhou, Z.; Zhu, W. A Typical Fungicide and Its Main Metabolite Promote Liver Damage in Mice through Impacting Gut Microbiota and Intestinal Barrier Function. *J. Agric. Food Chem.* **2021**, *69*, 13436–13447. https://doi.org/10.1021/acs.jafc.1c05508.
31. Salam, S.; Iqbal, Z.; Khan, A.A.; Mahmood, R. Oral administration of thiram inhibits brush border membrane enzymes, oxidizes proteins and thiols, impairs redox system and causes histological changes in rat intestine: A dose dependent study. *Pestic. Biochem. Physiol.* **2021**, *178*, 104915. https://doi.org/10.1016/j.pestbp.2021.104915.
